# Neurilemmoma of Tongue in A Young Female: A Case Report

**DOI:** 10.7759/cureus.47438

**Published:** 2023-10-21

**Authors:** Akash Doshi, Nitin Bhola

**Affiliations:** 1 Department of Oral and Maxillofacial Surgery, Sharad Pawar Dental College, Datta Meghe Institute of Higher Education and Research (DMIHER), Wardha, IND

**Keywords:** local anesthesia, surgical excision, benign tumour, schwannomas, neurilemmoma

## Abstract

Neurilemmomas are benign, slowly growing tumors originating from Schwann cells in peripheral nerves. The precise cause is unclear. They commonly occur in the head and neck region (25-48% of cases) and rarely in the oral cavity (1%). While lingual schwannomas can develop at any age, they are most frequently seen between the ages of 30 and 60 years. In this case, a 19-year-old female was diagnosed with a lingual schwannoma. She had experienced painless swelling along the left side of her tongue for two years. The examination revealed a non-tender, soft to firm, 2x1 cm lump on the left side of the tongue, covered by healthy mucosa, with no signs of cervical lymph node enlargement. The lesion was completely excised under local anesthesia for histopathological evaluation. Histological examination revealed spindle cells with slender, undulating nuclei in Antoni A and B regions. The prominent nuclear palisading feature typical of schwannomas was evident.

## Introduction

Neurilemmoma is a benign, slow-growing tumor originating from the neural sheaths of peripheral, cranial (excluding I and II), spinal, and autonomic nerves [1]. Neurolemmocytes produce a narrow border around each extracranial nerve fiber, enclosing more giant fibers in a protecting lining called a myelin sheath to boost nerve conductivity. Oligodendrocytes myelinate neurons after they depart the spinal cord and brain, followed by neurolemmocytes. Neurilemmoma develops when proliferating neurolemmocytes unite to produce the tumor load, including sensory and motor nerve fibers.

The etiology of the neurilemmoma is unknown. Many etiological variables have been identified, such as spontaneous growth, external damage, persistent pain, and radiation exposure [2]. The head and neck are involved in 25-48% of cases [3]. Schwannoma is the most prevalent nerve sheath tumor in the oral cavity, making up slightly over 1% of all reported benign tumors. The tongue is the most frequently affected, followed by other oral cavity soft tissue structures. It's worth noting that only about 50% of the tumors are associated with a nerve. Because of their proximity, it is difficult to differentiate between hypoglossal, glossopharyngeal, and lingual nerve origins on the tongue. The prevalence is highest within 30-60 years of age, with barely any preference for sex or gender [4].

The size and location of lesions determine the presence and severity of symptoms. In most cases, the swelling is asymptomatic, solitary, has a smooth surface, and grows slowly. Occasionally, it can lead to tenderness or irritation. The therapeutic objective is total excision, leading to a low chance of recurrence [5]. Malignant schwannomas are uncommon, occurring in 8-13.9% of cases [6].

## Case presentation

**Figure 1:**
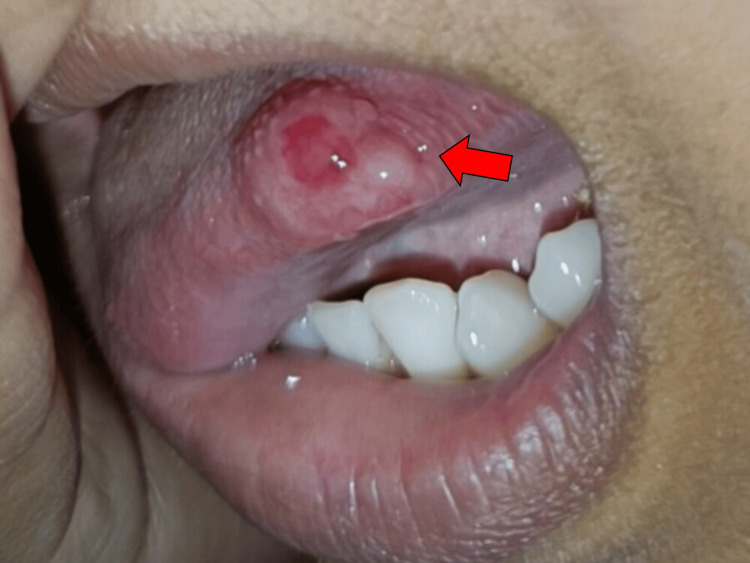
Asymptomatic nodule over the left lateral border of tongue.

A young girl sought examination at our department due to a slow-growing, infrequently tender swelling on the left side of her tongue, which she had noticed two years earlier. The patient's tongue was examined and found to be 2x1 cm in size. It was non-tender, soft to firm at the left lateral border, and covered by a normal mucous membrane (Figure 1).

**Figure 2:**
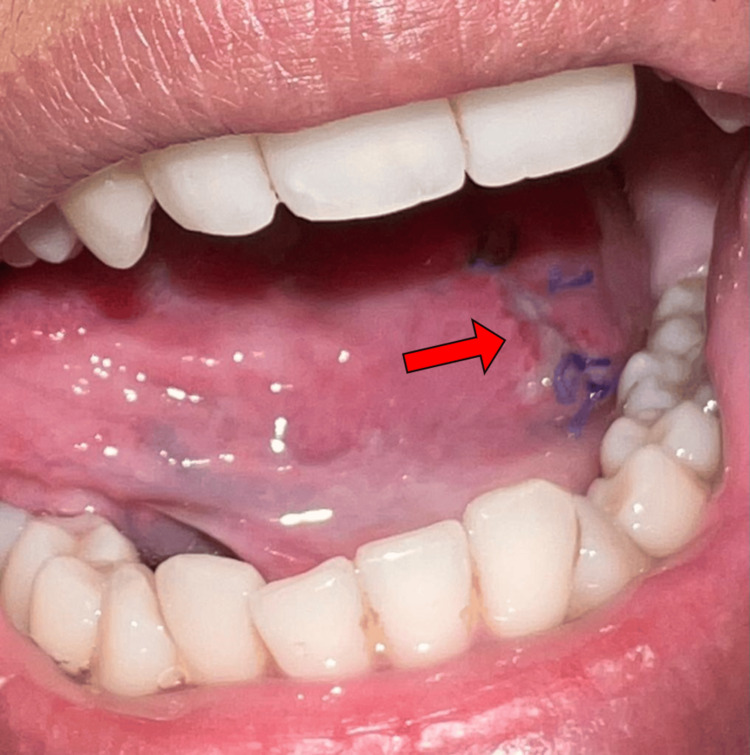
After the excision of the lesion.

There were no palpable lymph nodes in the neck, and the patient had no issues with eating, swallowing, phonation, or sensory/taste loss. Several possible diagnoses were considered, including traumatic fibroma, neurofibroma, and various benign lesions such as salivary gland tumors, leiomyoma, rhabdomyoma, lymphangioma, and hemangioma. Given the dimensions of the swelling, we planned to perform a complete excision of the lesion under local anesthesia. The tumor was found to be submucosal and was quickly removed by blunt dissection after creating a mucosal flap (Figure 2).

**Figure 3:**
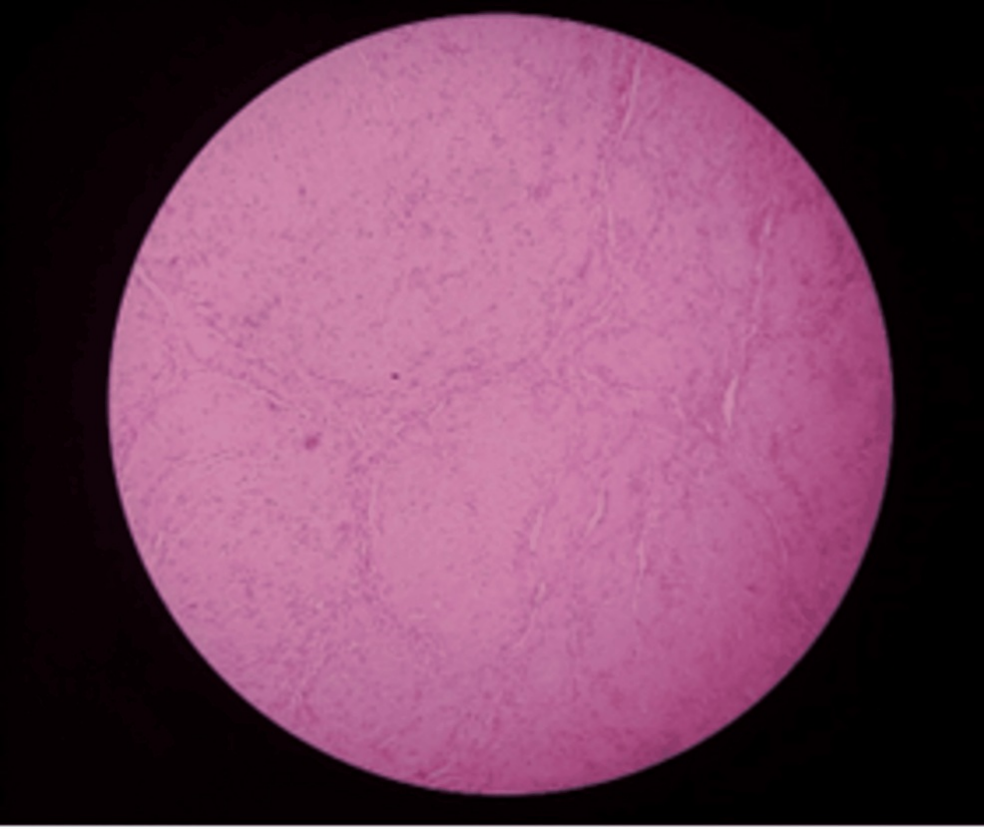
Histopathology image showing spindle-shaped neural cells arranged in Antoni A pattern with verocay bodies.

After the surgical removal of the lesion, a histopathological evaluation was conducted, which led to the diagnosis of neurilemmoma. The review identified the Antoni A pattern with verocay bodies and the Antoni B pattern (Figure 3). The patient underwent a one-year follow-up, and during this time, there was no recurrence of the condition.

It's important to note that neurilemmoma is a benign tumor that arises from Schwann cells of peripheral nerves. This case appears to have been successfully managed with surgical excision, and the patient's recovery was uneventful. Regular follow-ups are essential to monitor for any signs of recurrence.

## Discussion

Intraoral neurilemmomas are uncommon, comprising a mere 1% of all tumors found within the oral cavity. This remarkable rarity often leads to them being misdiagnosed during the initial diagnostic assessment of oral lesions. This underlines the importance of a comprehensive and thorough evaluation when diagnosing and treating oral conditions, as rare entities like neurilemmomas can present unique challenges and necessitate specialized approaches to management [7]. The clinical presentation of neurilemmomas can often resemble other encapsulated benign tumors within the oral cavity. The similarity in clinical features can make it challenging to definitively identify neurilemmomas based solely on physical examination and patient history. As a result, the gold standard for accurately diagnosing neurilemmomas and distinguishing them from other oral lesions is a biopsy followed by a thorough histological examination [8]. The primary treatment for neurilemmomas, as mentioned, is surgical excision. In this context, an excisional biopsy serves a dual purpose as a diagnostic and therapeutic procedure. The main objective of surgery in neurilemmomas is the complete removal of the tumor to prevent any potential recurrence. In situations where neurilemmomas are small and well-defined, such as in the described case, surgical procedures can be conducted with a relatively low risk of injury to nearby nerves or structures. It is imperative to exercise caution and precision, mainly when operating in the head and neck area, to preserve vital structures, particularly nerves [9]. The decision to perform a complete excision of the lesion is often made to minimize the risk of recurrence. By achieving complete tumor removal, the likelihood of residual tumor cells and the potential for regrowth are significantly reduced. This approach not only aids in securing a precise diagnosis but also contributes to a more favorable long-term prognosis for the patient [10].

The lesion described here had normal histology, with a thin fibrous capsule and a tumor-like development caused by Antoni types A and B tissue arrangements. Tissues of Antoni type A are densely packed, resulting in bundles with elongated, palisaded nuclei. Under an electron microscope, the so-called Verocay bodies appear composed of thin cytoplasmic processes with a small amount of collagen and basal laminar material. In contrast, the Antoni B tissue has fewer cells and less organization, with fusiform cells widely separated, dispersed loosely, and randomly with a network of delicate reticulata. The presence of nuclear palisading (a schwannoma characteristic) was seen. The acid S-100 protein test was skipped since the hematoxylin and eosin-stained sections confirmed the diagnosis.

The lesion described here exhibited normal histology, characterized by a thin fibrous capsule and a tumor-like structure resulting from the arrangement of Antoni types A and B tissues. Antoni type A tissue is characterized by densely packed cells, forming bundles with elongated nuclei arranged in a palisaded fashion. When examined under an electron microscope, these structures, known as Verocay bodies, appear to consist of thin cytoplasmic processes with a small amount of collagen and basal laminar material. In contrast, Antoni type B tissue is less organized, with fewer cells. The fusiform cells in Antoni B tissue are widely separated and dispersed loosely and randomly, often appearing within a network of delicate reticulata. Notably, nuclear palisading, a characteristic feature of schwannomas, was evident in the histological examination. In this case, the acid S-100 protein test was not performed, as the diagnosis was confirmed by examining hematoxylin and eosin-stained sections. These histological findings, including Antoni A and B tissue types and nuclear palisading, are consistent with diagnosing a neurilemmoma or schwannoma [11].

## Conclusions

Neurilemmomas in the tongue are relatively uncommon compared to other body parts with more abundant Schwann cells. These tumors tend to occur more frequently in middle-aged and older individuals, so cases in young patients are even rarer. As with many rare medical conditions, cases of tongue neurilemmomas may be underreported in the medical literature because they are not encountered frequently by healthcare professionals. A fresh case of tongue schwannoma is shown as an example of a lesion that is commonly missed or even identified as a probable diagnosis in clinical practice. A histopathological evaluation is needed for definitive diagnosis. The primary treatment for neurilemmomas, once diagnosed, is surgical removal. Surgical excision is often curative, and it helps prevent the lesion from recurring in most cases. The surgical approach may vary depending on the size and location of the tumor. Care should be taken to minimize damage to surrounding structures and nerves during the procedure.
